# Current burden, incidence trends, and future projections of pancreatic cancer in adults worldwide

**DOI:** 10.7189/jogh.16.04278

**Published:** 2026-08-28

**Authors:** Haiyan Guo, Yong Ling, Enhui Zhang, Shaona Li, Qin Zhao

**Affiliations:** 1 Department of Anesthesiology, The Affiliated Hospital of Qingdao University, Qingdao, China; 2 Department of Pharmacy, The Affiliated Hospital of Qingdao University, Qingdao, China

**Keywords:** adult pancreatic cancer, incidence, mortality, age-standardised rates, disease burden

## Abstract

**Background:**

Adult pancreatic cancer has a high mortality burden and poor survival. This study aimed to assess the current burden, geographic and socioeconomic differences, past incidence trends, and future burden.

**Methods:**

Publicly available data were obtained from the Global Cancer Observatory 2022 (GLOBOCAN 2022), Cancer Incidence in Five Continents Plus (CI5plus), the Global Burden of Disease study (GBD), Human Development Reports, the United Nations M49 classification, and World Population Prospects 2024. Adults aged 20 years and older were included. GLOBOCAN 2022 was used to estimate the disease burden in 2022. National Human Development Index values were correlated with age-standardised incidence rate (ASIR) and age-standardised mortality rate (ASMR) using Spearman correlation analysis. CI5plus data from 2000 to 2017 were used to assess sex-specific incidence trends using estimated annual percentage change. GBD data and World Population Prospects 2024 were used to project future burden from 2024 to 2045 using a Bayesian age-period-cohort model.

**Results:**

In 2022, there were 510,529 new cases and 467,290 deaths from pancreatic cancer worldwide among adults. Eastern Asia recorded the largest numbers of new cases and deaths. Western Europe had the highest ASIR and ASMR, while South Central Asia had the lowest. Countries with a very high Human Development Index (HDI) had the greatest burden, whereas those with a low HDI had the lowest. Adults aged 60–74 years accounted for the largest numbers of new cases and deaths. National Human Development Index was associated with ASIR and ASMR. Incidence trends from 2000 to 2017 varied across cancer registries and by sex. Projections showed increasing new cases and deaths from 2024 to 2045.

**Conclusions:**

Adult pancreatic cancer remains a major global health burden. The burden is projected to continue increasing. These findings may be useful for adult pancreatic cancer prevention in different regions.

Pancreatic cancer is less common than several other major cancers, but it has a very high mortality rate. Its mortality is close to its incidence in many populations, reflecting poor prognosis and limited survival. Recent Global Cancer Observatory (GLOBOCAN) reports have shown clear differences in pancreatic cancer incidence and mortality across countries and regions [1]. In addition, pancreatic neoplasms in children and adolescents are rare and differ in pathological characteristics, clinical presentation, and outcomes [2,3]. Therefore, investigating the global burden and regional variation of adult pancreatic cancer remains necessary.

Previous epidemiological studies have described marked geographic and socioeconomic differences in pancreatic cancer burden. Global studies have reported higher pancreatic cancer incidence and mortality in regions with higher socioeconomic development [4]. A study using Global Burden of Disease (GBD) data further showed that several areas in Europe, South America, and Greenland had relatively high incidence rates, supporting the presence of geographic variation in pancreatic cancer burden [5]. Recent analyses using GLOBOCAN 2022 also showed that pancreatic cancer incidence and mortality were positively associated with the Human Development Index (HDI) [6]. However, some studies mainly described current estimates, which limited the ability to assess observed temporal changes and future disease burden. Therefore, further analysis is needed to combine current burden estimates, regional differences, temporal trends, and future projections in adult pancreatic cancer.

The increasing availability of public cancer and population data sets provides an opportunity to assess adult pancreatic cancer burden from complementary perspectives. GLOBOCAN 2022 provides recent and comparable estimates of pancreatic cancer incidence and mortality across countries and regions, making it suitable for assessing the current global burden. When matched with national HDI data from the Human Development Reports, these estimates can be used to examine whether the burden differs according to socioeconomic development. Cancer Incidence in Five Continents Plus (CI5plus) contains long-term cancer registry data and can therefore be used to evaluate incidence trends [7]. In addition, GBD provides historical incidence, mortality, and population data, while World Population Prospects 2024 provides future population estimates. The combination of these data sources allows the present study to assess current burden, geographic variation, socioeconomic differences, temporal trends, and future incidence and mortality burden in adult pancreatic cancer.

Therefore, by using GLOBOCAN 2022, HDI level, CI5plus, GBD, and World Population Prospects 2024, this study aimed to assess the current burden and socioeconomic differences of adult pancreatic cancer, examine past incidence trends, and project future incidence and mortality burden worldwide.

**Adherence to JoGH’s Guidelines for Reporting Analyses of Big Data Repositories Open to the Public (GRABDROP)**

In accordance with JoGH’s Guidelines for Reporting Analyses of Big Data Repositories Open to the Public (GRABDROP) [8], detailed information on the originality of the study, prior related work using similar data repositories, statistical methods, and the use of artificial intelligence tools is provided (Table S1 in the **Online Supplementary Document**).

## METHODS

### Data sources and study population

This study used publicly available data from GLOBOCAN 2022, CI5plus, GBD, the Human Development Reports, the United Nations M49 classification, and World Population Prospects 2024.

GLOBOCAN 2022 data were accessed through the Global Cancer Observatory (Cancer Today) and were used to estimate the current incidence and mortality burden of adult pancreatic cancer in 2022 [9]. Data were extracted by countries, UN region, HDI level, and adult age group. Because the data sets used in this study report estimates in 5-year age groups and do not provide separate estimates for adults aged 18–19 years, the adult study population was defined as individuals aged 20 years and older, with 85+ years as the oldest age group. To describe the age distribution of adult pancreatic cancer burden, adults were further divided into four age groups: 20–44, 45–59, 60-74, and 75–85+ years. Data grouped by UN region and HDI level were downloaded directly from Cancer Today. In particular, the totals obtained by summing UN regions were not identical to the totals obtained by summing HDI levels.

The United Nations M49 classification was used to define world regions. National HDI values were obtained from the Human Development Reports and were matched with national ASIR and ASMR from GLOBOCAN 2022 for correlation analysis. CI5plus data were used to assess past incidence trends in adult pancreatic cancer by sex. GBD data were used to obtain historical incidence, mortality, and population data for projection analysis [10]. Future population estimates were obtained from World Population Prospects 2024 and were used together with GBD data to project future incidence and mortality burden.

### Statistical analysis

The current burden of adult pancreatic cancer in 2022 was described using the number of incident cases, number of deaths, age-standardised incidence rate (ASIR), age-standardised mortality rate (ASMR), mortality-to-incidence ratio (MIR), and cumulative risk. ASIR and ASMR were reported per 100,000 population. For GLOBOCAN 2022 estimates, 95% uncertainty intervals (UIs) were not available for the selected adult age range of 20–85+ years. In addition, MIR was calculated as the number of deaths divided by the number of incident cases. Mortality-to-incidence ratio was used as a descriptive indicator of the relationship between mortality and incidence. Because MIR can be affected by survival, cancer registration, death registration, and data completeness, it was not treated as a direct measure of treatment access or treatment effectiveness.

The association between national HDI and adult pancreatic cancer burden was assessed using Spearman correlation analysis. Bonferroni correction was applied, and statistical significance was assessed at *P* < 0.025 (0.05/2).

Past incidence trends in adult pancreatic cancer were assessed using CI5plus from 2000 to 2017. Analyses were conducted for males and females separately. The estimated annual percentage change (EAPC) was calculated, with 95% confidence intervals (CIs). Trends were classified as increasing, decreasing, or stable.

Future incidence and mortality burden of adult pancreatic cancer was projected using a Bayesian age-period-cohort (BAPC) model. Incidence, mortality, and population data from GBD were combined with future population estimates from World Population Prospects 2024 to project the number of incident cases, number of deaths, ASIR, and ASMR from 2024 to 2045. Projection results were reported with 95% UIs.

All analyses were performed using *R*, version 4.3.1 (R Foundation for Statistical Computing, Vienna, Austria). This study used publicly available aggregated data and did not involve individual-level information. Therefore, ethical approval and informed consent were not required.

## RESULTS

### Current burden of adult pancreatic cancer in 2022

In 2022, across UN regions, there were 510,529 incident cases and 467,290 deaths from adult pancreatic cancer. Among UN regions, Eastern Asia had the highest number of incident cases and deaths, with 177,069 cases and 158,998 deaths. Micronesia had the lowest number of incident cases and deaths, with 26 cases and 24 deaths. For age-standardised rates by UN region, Western Europe had the highest ASIR (14.7 per 100,000) and ASMR (13.2 per 100,000). South Central Asia had the lowest ASIR (2.0 per 100,000) and ASMR (1.9 per 100,000).

**Figure 1:**
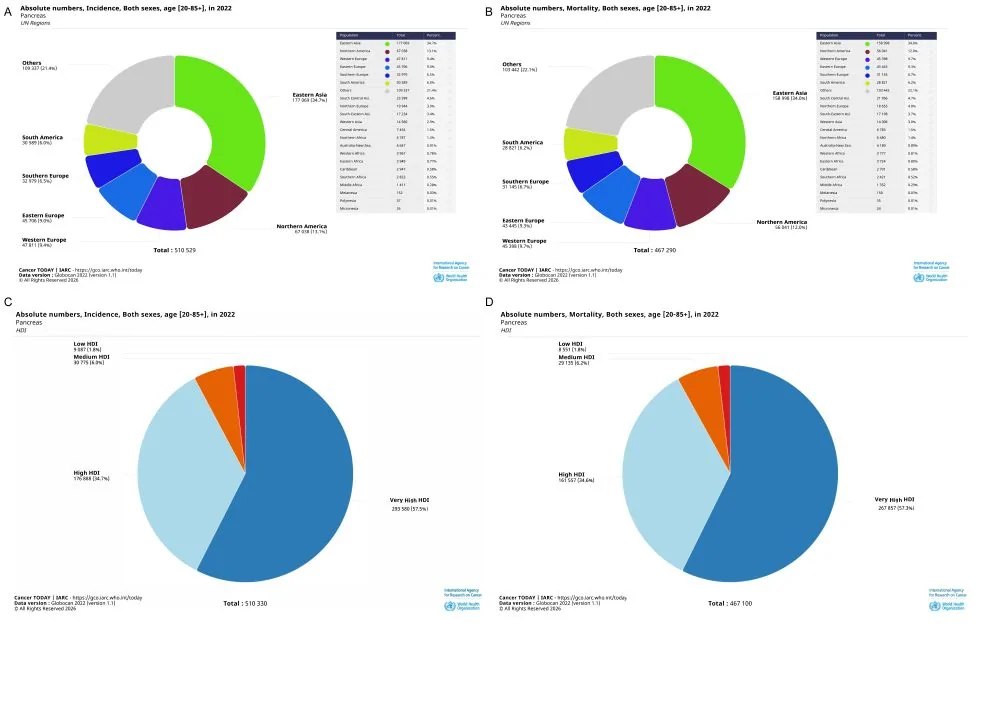
Distribution of adult pancreatic cancer cases and deaths by UN region and HDI level in 2022. Panel A. Incident cases by UN region. Panel B. Deaths by UN region. Panel C. Incident cases by HDI level. Panel D. Deaths by HDI level. HDI – Human Development Index, UN – United Nations.

**Figure 2:**
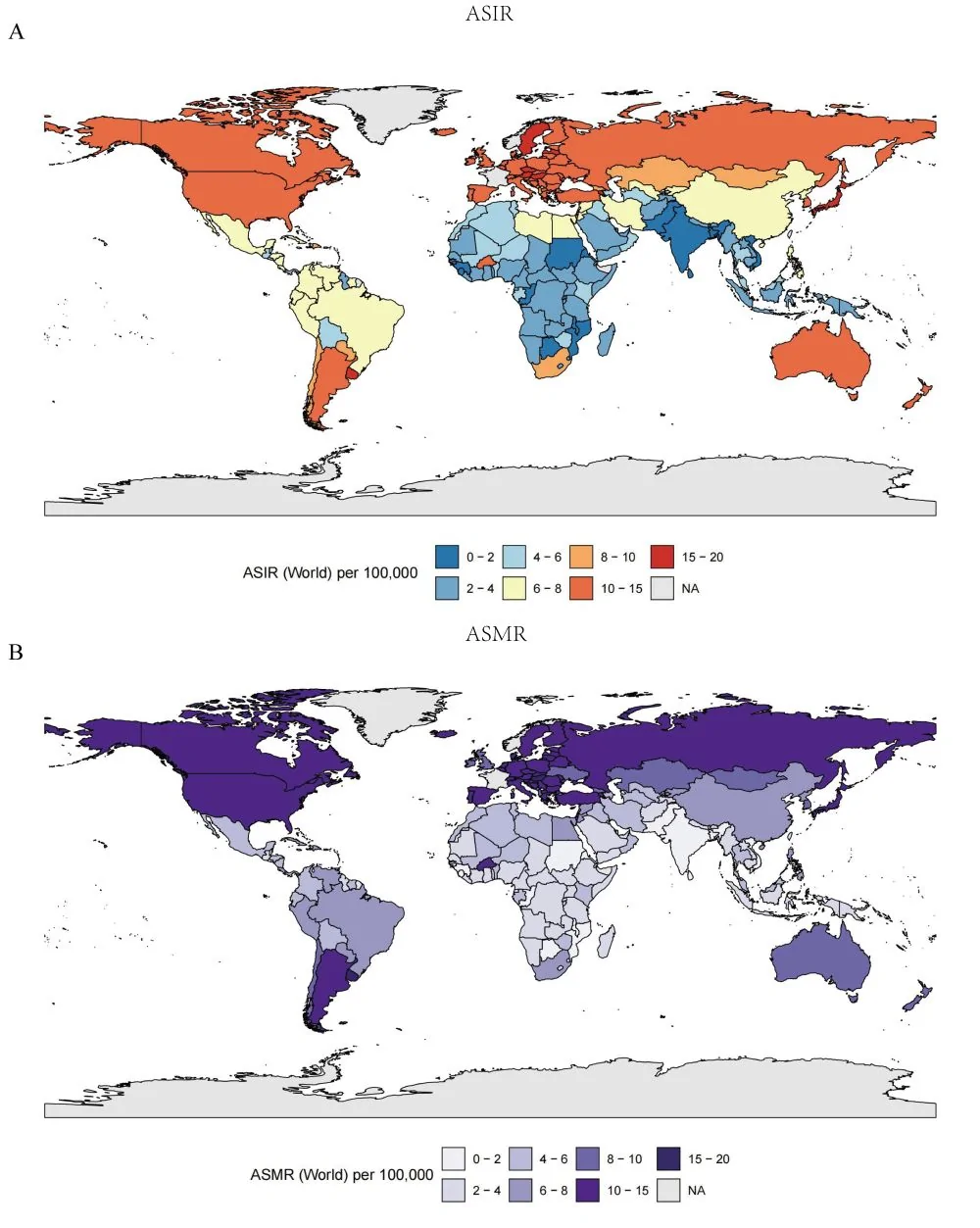
Global maps of adult pancreatic cancer incidence and mortality in 2022. Panel A. ASIR. Panel B. ASMR. ASIR – age-standardised incidence rate, ASMR – age-standardised mortality rate. Maps were generated using publicly available cancer burden data from the GLOBOCAN 2022 database.

By HDI level, very high HDI countries had the highest number of incident cases and deaths, with 293,580 cases and 267,857 deaths. Low HDI countries had the lowest number of incident cases and deaths, with 9087 cases and 8551 deaths. For age-standardised rates by HDI level, very high HDI countries had the highest ASIR (13.1 per 100,000) and ASMR (11.4 per 100,000). Low HDI countries had the lowest ASIR (2.3 per 100,000) and ASMR (2.2 per 100,000) (**Figure 1**, **Figure 2**, **Table 1**).

**Table 1:** Adult pancreatic cancer incidence and mortality in 2022 by regions and HDI level

	Incidence			Mortality		
	**Number**	**ASR**	**Cumulative risk**	**Number**	**ASR**	**Cumulative risk**
**UN regions**						
Northern America	67,038	14.1	1.00	56,041	11	0.78
Eastern Asia	177,069	8.8	0.59	158,998	7.6	0.50
Eastern Africa	3,949	2.9	0.21	3,724	2.7	0.20
Middle Africa	1,411	2.6	0.18	1,352	2.5	0.17
Northern Africa	6,787	5	0.36	6,480	4.8	0.35
Southern Africa	2,823	8.1	0.56	2,421	7.2	0.46
Western Africa	3,967	3.2	0.22	3,777	3.1	0.21
Caribbean	2,947	7.5	0.52	2,701	6.7	0.46
Central America	7,454	6.2	0.44	6,783	5.6	0.39
South-Eastern Asia	17,234	3.7	0.27	17,198	3.7	0.27
South Central Asia	23,399	2	0.15	21,956	1.9	0.14
Western Asia	14,560	9.1	0.63	14,006	8.8	0.60
Eastern Europe	45,706	12.7	0.95	43,445	11.8	0.88
Northern Europe	19,944	12.2	0.84	18,655	10.9	0.75
Southern Europe	32,979	12.8	0.89	31,145	11.4	0.79
Western Europe	47,811	14.7	1.00	45,398	13.2	0.92
Australia-New Zealand	4,647	11.3	0.78	4,180	9.7	0.65
Melanesia	152	3.5	0.22	150	3.5	0.21
South America	30,589	8.3	0.56	28,821	7.7	0.53
Micronesia	26	7.2	0.45	24	6.7	0.37
Polynesia	37	7.7	0.55	35	7.3	0.53
**HDI levels**						
Very High HDI	293,580	13.1	0.92	267,857	11.4	0.79
High HDI	176,888	6.9	0.49	161,557	6.2	0.43
Medium HDI	30,775	2.4	0.17	29,135	2.3	0.17
Low HDI	9,087	2.3	0.16	8,551	2.2	0.15

ASR – age-standardised rate, HDI – Human Development Index

The burden also differed by adult age group (**Table 2**). The highest numbers of both incident cases and deaths were observed among adults aged 60–74 years. Adults aged 20–44 years had the lowest number of incident cases and deaths.

**Table 2:** Adult pancreatic cancer incidence and mortality in 2022 by age group, UN region, and HDI level

	Incidence												Mortality											
	**20–44 years**			**45–59 years**			**60–74 years**			**75–85+ years**			**20–44 years**			**45–59 years**			**60–74 years**			**75–85+ years**		
	**Number**	**ASR**	**Cumulative risk**	**Number**	**ASR**	**Cumulative risk**	**Number**	**ASR**	**Cumulative risk**	**Number**	**ASR**	**Cumulative risk**	**Number**	**ASR**	**Cumulative risk**	**Number**	**ASR**	**Cumulative risk**	**Number**	**ASR**	**Cumulative risk**	**Number**	**ASR**	**Cumulative risk**
**UN regions**																								
Australia-New Zealand	79	0.69	0.02	562	9.1	0.15	1,841	37.7	0.61	2,165	90.4	0.65	49	0.42	0.01	426	6.9	0.11	1,571	31.9	0.53	2,134	88.8	0.61
Caribbean	113	0.71	0.02	583	7.1	0.11	1,179	23.7	0.38	1,072	52	0.4	58	0.37	0.01	502	6.1	0.1	1,084	21.8	0.36	1,057	51.2	0.38
Central America	349	0.49	0.01	1,567	5.4	0.09	3,201	20.7	0.34	2,337	42.9	0.34	257	0.36	0.01	1,374	4.7	0.08	2,908	18.8	0.31	2,244	41.2	0.32
Eastern Africa	461	0.32	0.01	1,121	2.8	0.05	1,693	9.6	0.15	674	16.4	0.13	359	0.25	0.01	1,052	2.7	0.04	1,639	9.2	0.15	674	16.3	0.13
Eastern Asia	3,520	0.57	0.02	29,563	7	0.11	72,807	28.7	0.46	71,179	71.6	0.5	2,322	0.37	0.01	22,806	5.4	0.09	62,945	24.5	0.4	70,925	71.1	0.46
Eastern Europe	1,050	0.86	0.02	8,284	13.6	0.22	23,562	44.6	0.71	12,810	63	0.59	748	0.59	0.02	7,398	12.1	0.2	22,328	42.2	0.67	12,971	63.4	0.57
Melanesia	9	0.23	0.01	48	3.3	0.05	62	9.6	0.16	33	32.1	0.17	9	0.23	0.01	46	3.2	0.05	60	9.3	0.15	35	34.5	0.17
Micronesia	0	0	0	3	3.4	0.05	13	24.4	0.4	10	81	0.47	0	0	0	3	3.4	0.05	11	20.8	0.32	10	81	0.39
Middle Africa	198	0.4	0.01	542	3.5	0.06	481	7.1	0.11	190	12.1	0.1	166	0.33	0.01	517	3.4	0.05	481	7.1	0.11	188	12	0.1
Northern Africa	512	0.53	0.01	1,962	5.7	0.09	3,001	16.2	0.26	1,312	26.2	0.23	429	0.45	0.01	1,829	5.3	0.08	2,922	15.8	0.25	1,300	26	0.22
Northern America	1,349	1	0.03	9,838	13.2	0.21	30,511	47.8	0.77	25,340	90.1	0.74	507	0.37	0.01	6,781	8.9	0.15	24,567	38.3	0.62	24,186	85.3	0.64
Northern Europe	242	0.63	0.02	2,249	9.8	0.16	7,555	41.1	0.67	9,898	97.8	0.71	82	0.21	0.01	1,757	7.5	0.12	7,050	38.2	0.62	9,766	96.3	0.69
Polynesia	0	0	0	9	7.3	0.12	16	25	0.43	12	64.8	0.57	0	0	0	7	5.5	0.1	16	25	0.43	12	64.8	0.57
South America	974	0.54	0.01	5,394	6.8	0.11	12,785	27	0.44	11,436	65.9	0.47	758	0.42	0.01	4,847	6.1	0.1	12,115	25.5	0.42	11,101	64	0.45
South Central Asia	2,010	0.25	0.01	6,751	2.2	0.03	10,861	6.7	0.11	3,777	8.8	0.09	1,631	0.2	0.01	6,475	2.1	0.03	10,006	6.2	0.1	3,844	9.1	0.08
South-Eastern Asia	1,010	0.37	0.01	3,936	3.2	0.05	8,030	12.6	0.21	4,258	24.6	0.21	918	0.33	0.01	3,717	3	0.05	8,190	12.9	0.21	4,373	25.3	0.21
Southern Africa	196	0.7	0.02	806	8.5	0.14	1,207	25.4	0.41	614	52.6	0.41	95	0.33	0.01	560	5.9	0.1	1,051	22.1	0.35	715	65.7	0.36
Southern Europe	374	0.67	0.02	4,355	11.6	0.19	12,175	42.6	0.69	16,075	93.3	0.7	197	0.33	0.01	3,424	9	0.15	11,145	38.8	0.63	16,379	94.2	0.67
Western Africa	481	0.4	0.01	1,348	3.6	0.05	1,550	9.5	0.15	588	19.6	0.15	434	0.36	0.01	1,233	3.3	0.05	1,533	9.4	0.15	577	19.4	0.15
Western Asia	735	0.61	0.02	3,292	7.6	0.12	6,332	30.5	0.49	4,201	68.2	0.49	482	0.4	0.01	2,989	6.9	0.11	6,151	29.6	0.48	4,384	71	0.5
Western Europe	517	0.78	0.02	5,787	12.7	0.21	18,331	50	0.81	23,176	107.6	0.81	229	0.33	0.01	4,632	10	0.16	16,896	45.9	0.75	23,641	109	0.78
**HDI levels**																								
Very High HDI	4,757	0.77	0.02	39,662	11.4	0.18	119,837	44.1	0.71	129,324	96.1	0.72	2,588	0.41	0.01	30,702	8.7	0.14	105,971	38.9	0.63	128,596	94.9	0.67
High HDI	5,810	0.53	0.01	36,772	6.1	0.1	80,070	23.3	0.38	54,236	47.6	0.38	4,218	0.38	0.01	30,719	5	0.08	72,015	20.9	0.34	54,605	47.9	0.36
Medium HDI	2,418	0.27	0.01	8,752	2.6	0.04	13,706	7.9	0.13	5,899	13.1	0.11	1,910	0.22	0.01	8,381	2.5	0.04	13,164	7.6	0.12	5,680	12.6	0.11
Low HDI	1,191	0.33	0.01	2,782	2.5	0.04	3,493	7	0.11	1,621	13.9	0.11	1,012	0.28	0.01	2,545	2.3	0.04	3,437	6.9	0.11	1,557	13.2	0.11

ASR – age-standardised rate, HDI – Human Development Index

### Geographic variation in national and regional age-standardised rates

**Figure 3:**
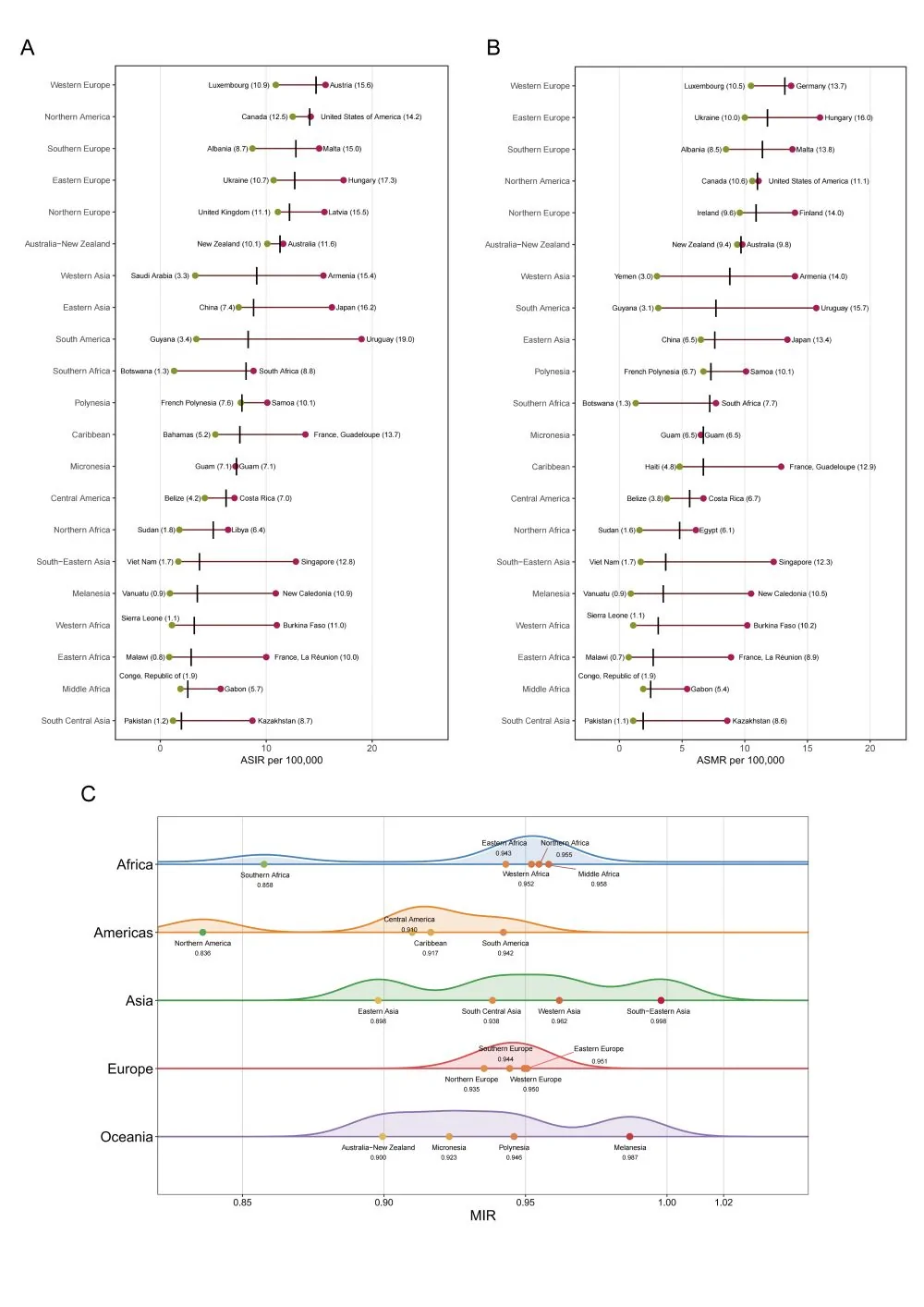
Geographic variation in adult pancreatic cancer burden in 2022. Panel A. Age-standardised incidence rates across countries and regions. Panel B. Age-standardised mortality rates across countries and regions. Panel C. Mortality-to-incidence ratio by region. ASIR – age-standardised incidence rate, ASMR– age-standardised mortality rate, MIR – mortality-to-incidence ratio.

Marked geographic variation in ASIR and ASMR was observed across countries and UN regions (**Figure 3**, Panels A–B). For ASIR, Uruguay had the highest national rate (19.0 per 100,000), whereas Malawi had the lowest national rate (0.8 per 100,000). For ASMR, Hungary had the highest national rate (16.0 per 100,000), whereas Malawi had the lowest national rate (0.74 per 100,000). At the regional level, Western Europe had the highest ASIR and ASMR, while South Central Asia had the lowest ASIR and ASMR.

MIR was high in most regions (**Figure 3**, Panel C). Northern America had the lowest MIR (0.836), whereas South-Eastern Asia had the highest MIR (0.998). These findings indicate that the number of deaths was close to the number of incident cases in many regions.

### Association between HDI and adult pancreatic cancer burden

National HDI was positively associated with both ASIR and ASMR of adult pancreatic cancer (Figure S1 in the **Online Supplementary Document**). The correlation between HDI and ASIR was significant (Spearman’s ρ = 0.793; *P* < 0.001). The correlation between HDI and ASMR was also significant (Spearman’s ρ = 0.788; *P* < 0.001). Both associations remained significant after Bonferroni correction. These results showed that countries with higher HDI generally had higher ASIR and ASMR.

### Past incidence trends from 2000 to 2017

Using CI5plus data from 2000 to 2017, EAPC analysis was stratified by cancer registry and sex (Figure S2 in the **Online Supplementary Document**; Table S2 in the **Online Supplementary Document**). Among males, the highest EAPC was observed in Thailand, Chiang Mai (EAPC = 5.99; 95% CI = 4.24, 7.77), whereas the lowest was observed in Poland, Kielce (EAPC = −3.00; 95% CI = −4.27, −1.72).

Among females, the highest EAPC was observed in France, Loire-Atlantique (EAPC = 6.96; 95% CI = 5.66, 8.28), whereas the lowest was observed in China, Nangang District, Harbin City (EAPC = −3.85; 95% CI = −5.28, −2.41).

### Future projections from 2024 to 2045

BAPC projections suggested that the future burden of adult pancreatic cancer would continue to increase from 2024 to 2045 (Figure S3 and Tables S3 and S4 in the **Online Supplementary Document**). The projected number of incident cases increased from 599,334 in 2024 to 1,336,141 in 2045, and ASIR increased from 9.37 to 11.52 per 100,000. The projected number of deaths increased from 572,526 in 2024 to 1,267,093 in 2045, and ASMR increased from 8.89 to 10.56 per 100,000. Projection results were reported with 95% UIs.

## DISCUSSION

This study provides a comprehensive assessment of adult pancreatic cancer, including current burden, past incidence trends, and future projections. Previous studies have shown that pancreatic cancer has a high mortality burden and marked geographic variation [11]. Consistent with these findings, our analysis showed that deaths were close to incident cases in many adult populations and varied across countries and regions. In addition, CI5plus showed that past incidence trends differed across registry populations and sex groups, while BAPC projections using GBD and future population estimates suggested that the future incidence and mortality burden may continue to increase. Together, these findings indicate that adult pancreatic cancer remains a major global health challenge. Several previous studies have examined the global burden of pancreatic cancer, including GLOBOCAN, GBD, and socioeconomic indicators such as HDI. These studies have described the global and regional incidence and mortality burden of pancreatic cancer, evaluated associations with socioeconomic development, mortality-to-incidence ratios, temporal trends, or future pancreatic cancer burden [4,6,7,11–18]. Building on these studies, this study further assessed adult pancreatic cancer burden.

The marked geographic differences and differences by HDI observed in this study are consistent with previous epidemiological reports. Earlier studies have shown that pancreatic cancer incidence and mortality are generally higher in more developed regions and vary across countries [19,20]. Recent analyses also reported positive associations between HDI and pancreatic cancer incidence and mortality [21]. In our study, countries with higher HDI generally had higher ASIR and ASMR, while large absolute numbers of cases and deaths were observed in regions with large and older populations. These findings may partly reflect differences in population ageing, smoking, obesity, diabetes, alcohol use, dietary habits, environmental and climatic conditions, genetic background, national health policies, treatment resources, and the completeness of cancer and death registration.

Previous studies reported higher pancreatic cancer incidence and mortality in more developed regions, including North America and Europe, and showed positive associations between socioeconomic development and pancreatic cancer burden [22,23]. Our results were generally consistent with these studies. Regions with large and ageing populations, such as Eastern Asia, contributed large absolute numbers of cases and deaths. For past trends, previous studies reported increasing pancreatic cancer incidence in several countries over recent decades [24–26]. Our EAPC analysis using CI5plus showed similar increases in some registry populations and sex groups. In addition, previous projection studies suggested that pancreatic cancer incidence or deaths may continue to rise [27–30]. Our BAPC projections were generally consistent with this direction and suggested a continued increase in future adult pancreatic cancer incidence and mortality. However, direct comparison with previous projections should be made cautiously because the present study focused on adults aged 20 years and older. Because pancreatic cancer burden is strongly concentrated in older adults [31,32], differences in the study population may affect projected case numbers, death numbers, and growth rates. Therefore, projections for adult pancreatic cancer remain necessary to better understand future burden in the population where most pancreatic cancer cases and deaths occur.

The highest number of incident cases and deaths was observed among adults aged 60–74 years. Although the risk of pancreatic cancer remains high after 75 years of age, the population aged 75–85+ years is smaller and is also affected by deaths from other causes. As a result, the absolute burden may peak among adults aged 60–74 years. In addition, the high MIR observed in most regions further emphasises the fatal nature of pancreatic cancer. In many populations, the number of deaths was close to the number of incident cases, which is consistent with poor prognosis and limited survival. From a public health perspective, this finding suggests that adult pancreatic cancer may continue to require substantial resources for diagnosis, treatment, and management.

These findings have several public health implications. The high current burden and projected future increase suggest that strengthened prevention and management of pancreatic cancer is needed, especially in regions with large and ageing adult populations. In countries with higher HDI, prevention and planning should account for population ageing and focus on metabolic disorders, smoking, alcohol use, obesity, and individuals at increased risk [33]. Although countries with low or medium HDI currently had lower ASIR and ASMR, risk factors may increase their future burden. This is particularly relevant for regions such as South Central Asia and parts of Sub-Saharan Africa, where an increasing adult and older population may create a greater need for pancreatic cancer prevention, diagnosis, and treatment. In countries with lower or medium HDI, strengthening cancer registration, death registration, and basic oncology services remains important for more accurate burden assessment. The high MIR further suggests that relying only on care after advanced disease has developed is unlikely to substantially reduce mortality. Greater attention should be given to risk factor control, identification of high-risk individuals, earlier recognition of disease, and support for patients with advanced cancer.

This study has several strengths. First, it focused on adult pancreatic cancer, the population in which most pancreatic cancer cases and deaths occur. Second, by combining GLOBOCAN 2022, CI5plus, and GBD, this study assessed the current burden, past incidence trends, and future incidence and mortality burden. Third, the study incorporated HDI level, which allowed assessment of socioeconomic differences in pancreatic cancer burden. Fourth, adult age groups were further divided into 20–44, 45–59, 60–74, and 75–85+ years, providing a clearer description of burden distribution across adult age groups.

This research has a number of limitations. First, estimates from public databases depend on the quality of cancer registration and death registration in each country, and data completeness may vary across countries and regions. Second, GLOBOCAN (Cancer Today) did not report 95% uncertainty intervals for the point estimates in the 20–85+ years age range used in this study. This limited our ability to assess the uncertainty around the GLOBOCAN estimates and the descriptive differences across countries and regions. Third, adults aged 18–19 years were not included because the available data sets report estimates in five-year age groups. Although pancreatic cancer is rare in this age range, this may have slightly underestimated the total adult burden. Fourth, MIR should be considered with caution because regional differences may reflect not only survival and treatment differences but also cancer registration, death registration, and data completeness. Finally, the BAPC projections did not directly incorporate future changes in risk factor prevalence, screening uptake, treatment advances, or behavioural trends, including changes in obesity, diabetes, smoking, alcohol use, and diet; therefore, future incidence and mortality may differ from our projections if these factors vary across countries and regions over time.

## CONCLUSIONS

In conclusion, adult pancreatic cancer remains a major global health burden, with deaths close to incident cases in many populations and clear differences across countries and regions. Higher ASIR and ASMR were generally observed in countries with higher HDI, while regions with large and ageing adult populations contributed a substantial absolute burden. Future incidence and mortality are projected to continue increasing. These findings support strengthened prevention and management of pancreatic cancer, especially in countries with high rates, regions with large ageing populations, and older adult groups. Greater attention should be given to risk factor control, identification of high-risk individuals, appropriate surveillance for eligible high-risk groups, earlier recognition of disease, and continued monitoring of disease burden.

## Additional material


Online Supplementary Document

## Data Availability

**Data availability:** The public data sources used in this study were accessed as follows: GLOBOCAN 2022/Cancer Today (https://gco.iarc.who.int/en), Human Development Reports/HDI (https://hdr.undp.org/data-center/documentation-and-downloads), United Nations M49 classification (https://unstats.un.org/unsd/methodology/m49/overview/), World Population Prospects 2024 (https://population.un.org/wpp/downloads?folder=Standard%20Projections&group=Population), CI5plus (https://ci5.iarc.fr/ci5plus/), and GBD (https://vizhub.healthdata.org/gbd-results/).
