# Supplementary material for: Current burden, incidence trends, and future projections of pancreatic cancer in adults worldwide
Source: J Glob Health. 2026 Aug 28;16:04278. doi: 10.7189/jogh.16.04278 (PMC13522901; doi:10.7189/jogh.16.04278)
Supplement: Supplementary file 1 [file jogh-16-04278-s001.pdf]

Supplment to: Guo H, Ling Y, Zhang E, Li S, Zhao Q. Current burden, incidence trends, and future projections of pancreatic cancer in adults worldwide. J Glob Health. 2026;16:04278.

Figure S1. Association between national HDI and adult pancreatic cancer burden in 2022. **Panel A.** Association between HDI and age-standardised incidence rate. **Panel B.** Association between HDI and age-standardised mortality rate.

ASIR– Age-standardised incidence rate; **ASMR–** Age-standardised mortality rate; **HDI–** Human Development Index.

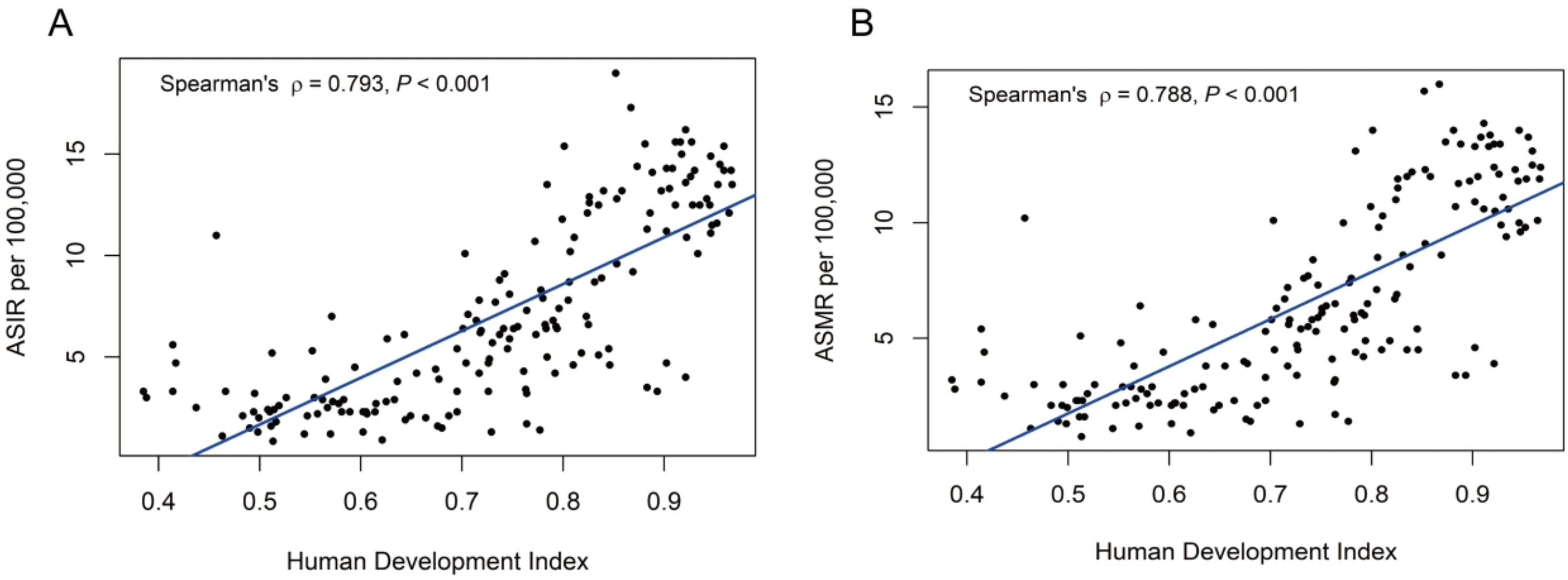

Figure S2. Past incidence trends of adult pancreatic cancer from 2000 to 2017 based on CI5plus data. **Panel A.** Estimated annual percentage change in age-standardised incidence rate among males. **Panel B.** Estimated annual percentage change in age-standardised incidence rate among females.

ASIR– Age-standardised incidence rate. **CI5plus** – Cancer Incidence in Five Continents Plus. **EAPC** – estimated annual percentage change.

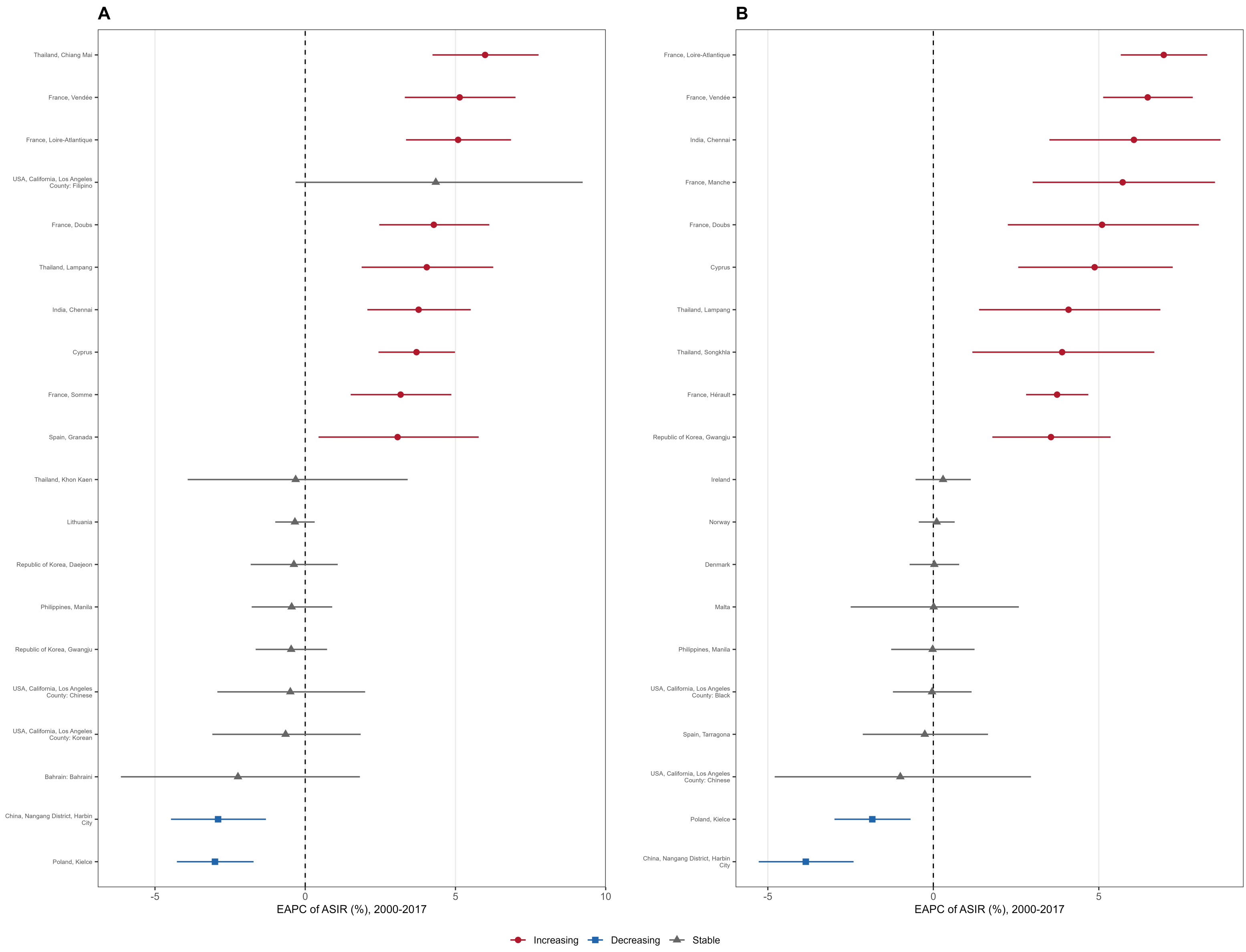

Figure S3. Projected age-standardised rates of adult pancreatic cancer from 2024 to 2045. **Panel A.** Projected ASIR. **Panel B.** Projected ASMR.
ASIR– Age-standardised incidence rate. **ASMR**– Age-standardised mortality rate.

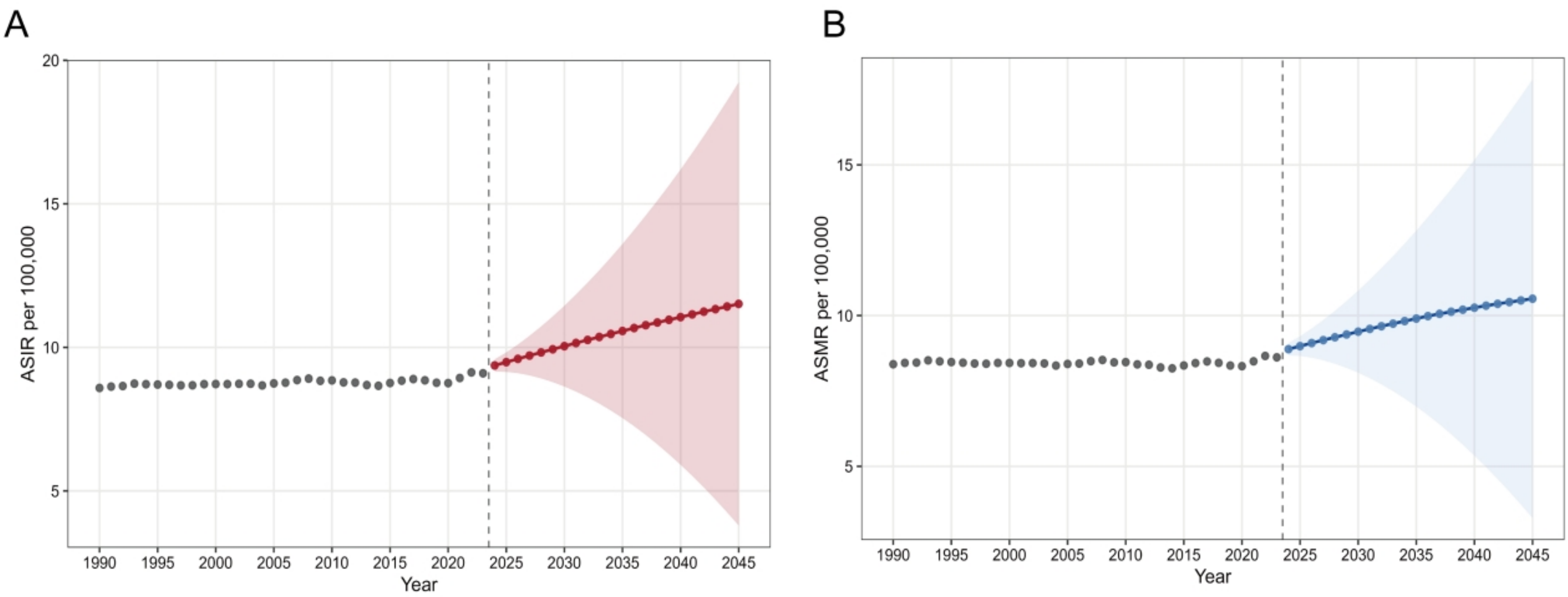

Table S1. Adherence to JoGH's GRABDROP guidelines.

JoGH guideline items

1. Please list all papers published by each co-author in previous 3 years that were based on secondary analysis of a big data repository

The authors reviewed the publication records of all co-authors for the previous three years. Qin Zhao published two original experimental studies during this period, both of which were basic laboratory studies and were not based on secondary analysis of a big data repository. No co-author has published, within the previous three years, a paper based on secondary analysis of a big data repository.

2. Please explain the key elements of your study design and the use of the available datasets that make your study an original scientific contribution

This study was designed to provide a comprehensive assessment of the current burden, regional differences, past trends, associations with HDI, and future projections of adult pancreatic cancer. GLOBOCAN 2022 data were used to estimate the current incidence and mortality burden among adults aged 20–85+ years, grouped by UN regions and HDI level. Country-level ASIR and ASMR were matched with national HDI values, and Spearman correlation analysis was used to examine the associations between HDI and ASIR or ASMR. CI5plus registry data from 2000 to 2017 were used to calculate the EAPC in ASIR separately for male and female. GBD historical incidence, mortality, and population data were combined with WPP 2024 future population data, and the BAPC model was used to project the number of incident cases, number of deaths, ASIR, and ASMR from 2024 to 2045. The originality of this study lies in the combined use of GLOBOCAN, CI5plus, GBD, World Population Prospects 2024, and HDI data to describe the current burden, regional differences, past trends, associations with HDI, and future burden of adult pancreatic cancer.

3. Please list all publications that addressed similar research questions in the same dataset and indicate where you cited them in your paper

Authors	PMID	Citation location
Wang, et al.	**40534262**	Introduction/Discussion
Zheng, et al.	**41614572**	Discussion
Huang, et al.	**33058868**	Discussion
Wong, et al.	**28600530**	Discussion
Goodarzi, et al.	**32660397**	Discussion
Sharma, et al.	**37818913**	Introduction/Discussion
Are, et al.	**27511902**	Discussion
Lee, et al.	**29982202**	Discussion
Veisani, et al.	**29408193**	Discussion
Vuković, et al.	**40800671**	Discussion
Ilic et al.	**36157927**	Introduction/Discussion
Amini et al.	**37471411**	Introduction

Publications were included in this table when they addressed pancreatic cancer using comparable public data sources and had an overall research scope closely aligned with the present study.

4. Please explain how you addressed multiple testing through an appropriately rigorous statistical threshold and indicate this in the methods section

Two correlation tests were performed: the association between HDI and ASIR and the association between HDI and ASMR. Bonferroni correction was applied, and statistical significance was assessed at $p < 0.025$ (0.05/2). EAPC was used only for trend analysis and was reported with 95% confidence intervals. BAPC results were reported with 95% uncertainty intervals.
5. Please declare to what extent have AI chatbots been used in developing your paper and to which parts of the paper did they contribute
ChatGPT was used only for auxiliary editorial support, including helping the authors understand reviewers' comments, improving wording, correcting spelling and grammatical errors, improving clarity and readability, and assisting with consistency checks of figures and tables. The AI did not contribute to study conception, data collection, statistical analysis, figure or table generation, assessment of results, or scientific conclusions. All authors reviewed, verified, and approved the final content of the manuscript.

ASIR – age-standardised incidence rate; **ASMR** – age-standardised mortality rate; **BAPC** – Bayesian age-period-cohort; **CI5plus** – Cancer Incidence in Five Continents Plus; **EAPC** – estimated annual percentage change; **GBD** – Global Burden of Disease; **GRABDROP** – Guidelines for Reporting Analyses of Big Data Repositories Open to the Public; **HDI** – Human Development Index; **JoGH** – Journal of Global Health; **PMID** – PubMed identifier; **WPP** – World Population Prospects.

Table S2. Estimated annual percentage changes in adult pancreatic cancer incidence from 2000 to 2017 by cancer registry and sex.

Registry population	sex	EAPC (95% CI)
Australia, New South Wales & Australian Capital Territory	Female	2.138 (1.674 to 2.605)*
Australia, New South Wales & Australian Capital Territory	Male	2.005 (1.548 to 2.463)*
Australia, Northern Territory	Female	2.738 (-2.951 to 8.76)
Australia, Northern Territory	Male	2.116 (-3.357 to 7.899)
Australia, Queensland	Female	1.393 (0.744 to 2.046)*
Australia, Queensland	Male	1.085 (0.343 to 1.832)*
Australia, South	Female	2.426 (1.259 to 3.606)*
Australia, South	Male	0.945 (-0.433 to 2.343)
Australia, Tasmania	Female	2.839 (-0.164 to 5.932)
Australia, Tasmania	Male	1.312 (-0.76 to 3.426)
Australia, Victoria	Female	1.22 (0.367 to 2.08)*
Australia, Victoria	Male	0.535 (-0.259 to 1.335)
Australia, Western	Female	0.764 (-0.346 to 1.888)
Australia, Western	Male	1.536 (0.508 to 2.573)*
Austria, Tyrol	Female	2.668 (1.069 to 4.292)*
Austria, Tyrol	Male	1.466 (-0.119 to 3.076)
Austria, Vorarlberg	Female	1.244 (-0.758 to 3.286)
Austria, Vorarlberg	Male	1.454 (-0.736 to 3.691)
Austria	Female	0.965 (0.432 to 1.501)*
Austria	Male	0.912 (0.438 to 1.389)*
Bahrain: Bahraini	Female	0.531 (-3.844 to 5.106)
Bahrain: Bahraini	Male	-2.238 (-6.135 to 1.82)
Belarus	Female	1.784 (1.063 to 2.511)*
Belarus	Male	1.988 (1.593 to 2.385)*
Canada (Excl Nova Scotia, Northwest Territories, Nunavut, Quebec and Yukon)	Female	1.04 (0.618 to 1.463)*
Canada (Excl Nova Scotia, Northwest Territories, Nunavut, Quebec and Yukon)	Male	1.205 (0.65 to 1.763)*
Chile, Valdivia	Female	2.616 (-2.571 to 8.078)

Chile, Valdivia	Male	0.467 (-2.889 to 3.939)
China, Jiashan County	Female	1.415 (-1.019 to 3.91)
China, Jiashan County	Male	2.021 (0.565 to 3.499)*
China, Nangang District, Harbin City	Female	-3.853 (-5.276 to -2.41)*
China, Nangang District, Harbin City	Male	-2.9 (-4.467 to -1.307)*
China, Zhongshan City	Female	1.975 (-0.581 to 4.596)
China, Zhongshan City	Male	3.009 (1.475 to 4.566)*
Colombia, Cali	Female	0.385 (-0.893 to 1.678)
Colombia, Cali	Male	1.56 (-0.099 to 3.247)
Croatia	Female	1.436 (0.86 to 2.016)*
Croatia	Male	0.066 (-0.514 to 0.65)
Cyprus	Female	4.873 (2.565 to 7.233)*
Cyprus	Male	3.703 (2.438 to 4.984)*
Czech Republic	Female	0.868 (0.517 to 1.219)*
Czech Republic	Male	0.211 (-0.128 to 0.551)
Denmark	Female	0.03 (-0.716 to 0.781)
Denmark	Male	0.511 (-0.066 to 1.091)
Ecuador, Quito	Female	1.713 (-0.09 to 3.549)
Ecuador, Quito	Male	1.337 (-0.637 to 3.35)
Estonia	Female	1.863 (0.556 to 3.188)*
Estonia	Male	0.418 (-0.449 to 1.293)
France, Calvados	Female	1.312 (0.192 to 2.444)*
France, Calvados	Male	0.117 (-1.385 to 1.641)
France, Doubs	Female	5.098 (2.251 to 8.023)*
France, Doubs	Male	4.279 (2.464 to 6.127)*
France, Haut-Rhin	Female	3.497 (1.6 to 5.43)*
France, Haut-Rhin	Male	2.655 (1.322 to 4.006)*
France, Hérault	Female	3.739 (2.804 to 4.683)*
France, Hérault	Male	2.851 (1.517 to 4.203)*
France, Isère	Female	3.186 (1.838 to 4.552)*
France, Isère	Male	2.703 (1.297 to 4.129)*
France, Loire-Atlantique	Female	6.962 (5.664 to 8.277)*
France, Loire-Atlantique	Male	5.087 (3.354 to 6.849)*
France, Manche	Female	5.72 (3.002 to 8.51)*
France, Manche	Male	2.85 (0.995 to 4.739)*
France, Martinique	Female	1.659 (-1.229 to 4.631)
France, Martinique	Male	2.075 (-0.307 to 4.513)
France, Somme	Female	2.678 (-0.025 to 5.454)
France, Somme	Male	3.174 (1.513 to 4.862)*
France, Vendée	Female	6.477 (5.132 to 7.839)*
France, Vendée	Male	5.14 (3.315 to 6.998)*

Germany, Hamburg	Female	1.384 (0.732 to 2.04)*
Germany, Hamburg	Male	1.058 (0.351 to 1.771)*
Germany, Saarland	Female	0.769 (-1.08 to 2.652)
Germany, Saarland	Male	0.304 (-1.043 to 1.668)
Iceland	Female	1.957 (-0.551 to 4.53)
Iceland	Male	0.151 (-3.074 to 3.483)
India, Chennai	Female	6.06 (3.508 to 8.676)*
India, Chennai	Male	3.774 (2.068 to 5.507)*
India, Mumbai	Female	2.895 (1.58 to 4.227)*
India, Mumbai	Male	2.841 (1.804 to 3.888)*
Ireland	Female	0.294 (-0.537 to 1.132)
Ireland	Male	0.449 (-0.491 to 1.398)
Israel: All Jews	Female	0.599 (-0.035 to 1.237)
Israel: All Jews	Male	0.261 (-0.143 to 0.666)
Israel	Female	0.297 (-0.343 to 0.94)
Israel	Male	-0.031 (-0.422 to 0.362)
Italy, Syracuse	Female	1.161 (-0.628 to 2.982)
Italy, Syracuse	Male	1.19 (-0.896 to 3.32)
Japan, Miyagi Prefecture	Female	1.79 (0.845 to 2.744)*
Japan, Miyagi Prefecture	Male	1.033 (0.472 to 1.597)*
Kuwait: Kuwaiti	Female	1.226 (-2.231 to 4.806)
Kuwait: Kuwaiti	Male	1.698 (-1.682 to 5.194)
Latvia	Female	1.577 (0.891 to 2.267)*
Latvia	Male	0.793 (-0.022 to 1.614)
Lithuania	Female	1.284 (0.679 to 1.892)*
Lithuania	Male	-0.343 (-0.997 to 0.315)
Malta	Female	0.011 (-2.497 to 2.584)
Malta	Male	1.553 (-0.486 to 3.634)
New Zealand	Female	1.804 (1.092 to 2.521)*
New Zealand	Male	1.263 (0.657 to 1.872)*
Norway	Female	0.101 (-0.44 to 0.645)
Norway	Male	0.016 (-0.565 to 0.6)
Philippines, Manila	Female	-0.022 (-1.273 to 1.246)
Philippines, Manila	Male	-0.451 (-1.781 to 0.897)
Poland, Kielce	Female	-1.844 (-2.987 to -0.688)*
Poland, Kielce	Male	-3.004 (-4.272 to -1.719)*
Republic of Korea, Busan	Female	2.557 (1.484 to 3.641)*
Republic of Korea, Busan	Male	1.352 (0.363 to 2.351)*
Republic of Korea, Daegu	Female	1.852 (0.915 to 2.796)*
Republic of Korea, Daegu	Male	1.357 (0.185 to 2.542)*
Republic of Korea, Daejeon	Female	2.72 (0.636 to 4.847)*
Republic of Korea,	Male	-0.377 (-1.815 to 1.082)

Daejeon		
Republic of Korea, Gwangju	Female	3.554 (1.784 to 5.356)*
Republic of Korea, Gwangju	Male	-0.468 (-1.649 to 0.728)
Republic of Korea, Incheon	Female	2.125 (1.062 to 3.199)*
Republic of Korea, Incheon	Male	0.349 (-0.989 to 1.705)
Republic of Korea, Seoul	Female	1.72 (1.03 to 2.415)*
Republic of Korea, Seoul	Male	0.355 (-0.255 to 0.969)
Republic of Korea, Ulsan	Female	1.556 (-0.031 to 3.168)
Republic of Korea, Ulsan	Male	1.176 (-0.4 to 2.777)
Republic of Korea	Female	2.344 (2.123 to 2.565)*
Republic of Korea	Male	0.643 (0.424 to 0.862)*
Slovenia	Female	1.153 (0.104 to 2.214)*
Slovenia	Male	0.745 (-0.193 to 1.691)
Spain, Girona	Female	1.201 (-0.47 to 2.901)
Spain, Girona	Male	0.546 (-0.882 to 1.996)
Spain, Granada	Female	1.921 (0.324 to 3.543)*
Spain, Granada	Male	3.074 (0.444 to 5.773)*
Spain, Murcia	Female	3.382 (2.088 to 4.692)*
Spain, Murcia	Male	1.648 (0.456 to 2.854)*
Spain, Tarragona	Female	-0.258 (-2.132 to 1.652)
Spain, Tarragona	Male	2.496 (0.712 to 4.313)*
Switzerland, Geneva	Female	0.816 (-0.703 to 2.359)
Switzerland, Geneva	Male	2.293 (0.213 to 4.416)*
Switzerland, Graubünden and Glarus	Female	1.734 (-1.408 to 4.977)
Switzerland, Graubünden and Glarus	Male	-0.221 (-3.05 to 2.692)
Switzerland, Ticino	Female	0.346 (-1.257 to 1.976)
Switzerland, Ticino	Male	0.769 (-1.755 to 3.357)
Switzerland, Valais	Female	1.389 (-0.9 to 3.732)
Switzerland, Valais	Male	0.419 (-2.285 to 3.197)
Switzerland, Vaud	Female	2.872 (1.121 to 4.654)*
Switzerland, Vaud	Male	0.942 (-0.217 to 2.114)
Thailand, Chiang Mai	Female	3.023 (-0.813 to 7.007)
Thailand, Chiang Mai	Male	5.986 (4.236 to 7.766)*
Thailand, Khon Kaen	Female	2.382 (-0.654 to 5.511)
Thailand, Khon Kaen	Male	-0.318 (-3.912 to 3.409)
Thailand, Lampang	Female	4.085 (1.382 to 6.86)*
Thailand, Lampang	Male	4.045 (1.878 to 6.257)*

Thailand, Songkhla	Female	3.891 (1.179 to 6.677)*
Thailand, Songkhla	Male	2.75 (-1.449 to 7.128)
The Netherlands	Female	2.361 (1.898 to 2.826)*
The Netherlands	Male	1.487 (1.049 to 1.926)*
Türkiye, Antalya	Female	2.419 (0.813 to 4.051)*
Türkiye, Antalya	Male	2.845 (0.688 to 5.048)*
Türkiye, Izmir	Female	2.727 (1.562 to 3.906)*
Türkiye, Izmir	Male	2.575 (1.642 to 3.517)*
UK, England	Female	1.326 (1.114 to 1.538)*
UK, England	Male	1.041 (0.855 to 1.228)*
UK, Northern Ireland	Female	1.349 (0.314 to 2.395)*
UK, Northern Ireland	Male	1.448 (-0.179 to 3.102)
UK, Scotland	Female	0.784 (0.292 to 1.277)*
UK, Scotland	Male	0.457 (-0.051 to 0.967)
USA, California, Los Angeles (SEER)	Female	0.542 (0.111 to 0.975)*
USA, California, Los Angeles (SEER)	Male	0.689 (0.253 to 1.126)*
USA, California, Los Angeles County: Black	Female	-0.039 (-1.221 to 1.156)
USA, California, Los Angeles County: Black	Male	-0.069 (-1.289 to 1.165)
USA, California, Los Angeles County: Chinese	Female	-0.997 (-4.793 to 2.95)
USA, California, Los Angeles County: Chinese	Male	-0.495 (-2.925 to 1.995)
USA, California, Los Angeles County: Filipino	Female	0.678 (-1.424 to 2.825)
USA, California, Los Angeles County: Filipino	Male	4.345 (-0.325 to 9.233)
USA, California, Los Angeles County: Hispanic White	Female	1.176 (0.208 to 2.153)*
USA, California, Los Angeles County: Hispanic White	Male	1.279 (0.146 to 2.424)*
USA, California, Los Angeles County: Korean	Female	1.999 (-1.225 to 5.328)
USA, California, Los Angeles County: Korean	Male	-0.653 (-3.091 to 1.846)
USA, California, Los Angeles County: Non-Hispanic White	Female	0.408 (-0.101 to 0.919)

USA, California, Los Angeles County: Non-Hispanic White	Male	0.757 (0.224 to 1.292)*
USA, California, Los Angeles County: White	Female	0.825 (0.32 to 1.333)*
USA, California, Los Angeles County: White	Male	0.832 (0.329 to 1.337)*
USA, NPCR (45 States and Washington DC)	Female	1.236 (1.131 to 1.342)*
USA, NPCR (45 States and Washington DC)	Male	1.154 (1.086 to 1.222)*
USA, NPCR: Asian and Pacific Islander	Female	0.65 (0.321 to 0.981)*
USA, NPCR: Asian and Pacific Islander	Male	0.709 (0.228 to 1.193)*
USA, NPCR: Black	Female	1.048 (0.809 to 1.289)*
USA, NPCR: Black	Male	0.78 (0.464 to 1.097)*
USA, NPCR: White	Female	1.288 (1.176 to 1.401)*
USA, NPCR: White	Male	1.23 (1.154 to 1.306)*
USA, SEER (9 registries): Black	Female	0.453 (-0.306 to 1.218)
USA, SEER (9 registries): Black	Male	0.283 (-0.541 to 1.113)
USA, SEER (9 registries): White	Female	1.065 (0.775 to 1.355)*
USA, SEER (9 registries): White	Male	0.783 (0.503 to 1.064)*
USA, SEER (9 registries)	Female	0.975 (0.69 to 1.261)*
USA, SEER (9 registries)	Male	0.732 (0.473 to 0.992)*
Uganda, Kyadondo County	Male	1.326 (-3.412 to 6.296)

* Statistically significant trend; **CI** − confidence interval; **EAPC** − estimated annual percentage change.

Table S3. Projected incidence burden of adult pancreatic cancer from 2024 to 2045.

Year	Predicted_incident_cases	Predicted_incident_cases_lower	Predicted_incident_cases_upper	ASIR	ASIR_lower	ASIR_upper
2024	599334	573654	625014	9.37	9.155	9.585
2025	624077	592302	655852	9.485	9.146	9.824
2026	649811	608566	691055	9.6	9.100	10.101
2027	676907	622930	730883	9.714	9.024	10.404
2028	705347	635511	775182	9.824	8.919	10.730
2029	734551	645817	823285	9.934	8.788	11.079
2030	764544	653899	875189	10.043	8.635	11.450
2031	795328	659712	930944	10.152	8.460	11.844
2032	827311	663473	991149	10.259	8.262	12.256
2033	860895	665295	1056496	10.363	8.041	12.685
2034	895396	664393	1126399	10.467	7.799	13.134
2035	930659	660486	1200831	10.57	7.537	13.604
2036	966456	653229	1279683	10.674	7.254	14.093
2037	1002974	642529	1363418	10.773	6.949	14.596
2038	1040772	628454	1453090	10.867	6.622	15.112
2039	1079241	610295	1548186	10.96	6.274	15.645
2040	1118409	587816	1649003	11.054	5.908	16.199
2041	1158466	560809	1756123	11.149	5.524	16.775
2042	1199915	529116	1870714	11.243	5.120	17.365
2043	1243765	492629	1994901	11.333	4.696	17.969
2044	1289276	450401	2128150	11.423	4.253	18.592
2045	1336141	401771	2270510	11.515	3.792	19.239

ASIR - Age-standardised incidence rate per 100,000 population.

Table S4. Projected mortality burden of adult pancreatic cancer from 2024 to 2045.

Year	Predicted_deaths	Predicted_deaths_lower	Predicted_deaths_upper	ASMR	ASMR_lower	ASMR_upper
2024	572526	548119	596934	8.885	8.679	9.092
2025	596171	565702	626639	8.986	8.659	9.314
2026	620661	580809	660512	9.087	8.603	9.571
2027	646296	593892	698699	9.185	8.517	9.854
2028	673134	605149	741119	9.28	8.403	10.157
2029	700630	614122	787137	9.373	8.264	10.482
2030	728780	620844	836717	9.465	8.104	10.827
2031	757765	625421	890109	9.558	7.922	11.193
2032	788082	628140	948025	9.647	7.719	11.575
2033	820007	628995	1011019	9.732	7.493	11.971
2034	852826	627181	1078471	9.816	7.247	12.386
2035	886389	622419	1150360	9.900	6.982	12.819
2036	920404	614322	1226485	9.982	6.697	13.267
2037	955086	602824	1307347	10.058	6.391	13.725
2038	991051	588020	1394082	10.127	6.063	14.192
2039	1027694	569202	1486187	10.194	5.717	14.672
2040	1065019	546116	1583922	10.261	5.353	15.168
2041	1103015	518457	1687574	10.327	4.974	15.681
2042	1141946	485974	1797918	10.390	4.576	16.204
2043	1182579	448501	1916658	10.447	4.160	16.734
2044	1224299	405227	2043371	10.503	3.728	17.277
2045	1267093	355659	2178526	10.559	3.281	17.837

ASMR – Age-standardised mortality rate per 100,000 population
